# Identification of caries-active individuals in longitudinal data a methodological study using a national registry

**DOI:** 10.1080/00016357.2023.2265474

**Published:** 2024-03-26

**Authors:** Håkan Flink, Anders Hedenbjörk-Lager, Simon Liljeström, Eva Nohlert, Åke Tegelberg

**Affiliations:** a Region Vastmanland, Uppsala University, Centre for Clinical Research, Vastmanland Hospital Vasteras, Vasteras, Sweden; b Faculty of Odontology, Malmö University, Malmö, Sweden

**Keywords:** Caries experience, caries prevention, dental care plan, disease progression, epidemiology

## Abstract

**Objective:**

The aim was to identify caries active individuals among adults by using a trajectory model of longitudinal data from the Swedish national registry (SKaPa) and comparing them with published data from the Dunedin cohort.

**Materials and methods:**

Data from two different age groups (30- and 40-year-olds) followed for 10 years were retrieved from SKaPa and were compared with published longitudinal birth-cohort data from the Dunedin study. Using the trajectory model, the subjects were divided into three different trajectories according to their caries development over time (i.e. high, 15%; moderate, 45%; low, 40%).

**Results:**

Caries experience, as measured by mean decayed, missing, and filled surfaces (DMFS) index, revealed significant differences among the three trajectories in both age groups. The patterns were similar to those observed in the Dunedin cohort. The mean increase in DMFS during the 10-year follow-up period from SKaPa was significantly higher for the high trajectories in both age groups compared with the moderate and low trajectories.

**Conclusions:**

The method using three trajectories for presentation of caries experience over time, may be a useful tool to identify subjects with different disease activities. Identification of subjects in the high caries experience trajectory may increase the possibility to explore and evaluate more effective caries prevention for this group in the future.

## Introduction

Dental caries in the permanent dentition is the most prevalent noncommunicable disease worldwide [1,2]. Although cross-sectional as well as longitudinal studies describe caries experience among children and teenagers [3], similar studies for adults are rare, even though the disease continues to affect patients throughout adulthood. The available data indicate that individuals with active caries disease continue to be caries active for many years [4–7]. However, national oral health surveys are complex and costly to conduct, and therefore seldom performed [2]. This lack of up-to-date epidemiologic data limits the development of appropriate approaches to reduce the burden of disease imposed by dental caries [2,8].

The most common method to describe caries experience is by using mean decayed, missing, and filled surfaces (DMFS) index values. Unfortunately, such data may hide the complexity of skewed distributions among those individuals with the most pronounced experience of disease, especially as mean caries experience (expressed by DMFS index values) among adults has decreased over time in many countries [9–11], resulting in an increasingly uneven distribution in the population data [8]. The proportion of individuals with no or few caries lesions is increasing. Thus, a minority with recurring caries disease will be obscured by lower mean DMFS values [8].

The largest longitudinal birth cohort of caries experience among adults is the Dunedin (New Zealand) cohort, which began in 1972 [12,13]. The observed population was divided into three different caries development trajectories [4,7].The group with the greatest caries experience (15%) presented with recurring caries disease, whereas 45% presented with low or limited caries development, while 40% had almost no caries development. The obvious difference in mean DMFS index between the described trajectories provides important information about the differences in caries experience.

Some main findings in studies of this population have demonstrated a strong relationship between socioeconomic inequality and caries prevalence and tooth loss [14]. The Dunedin cohort has been described in several scientific articles concerning oral health [12]. Some findings have been that a mother’s oral health reflects her children’s caries experiences later in life [15] and that unpleasant dental experiences may lead to the development of dental fear later in life [16].

The Swedish Quality Registry for Caries and Periodontal Diseases (SKaPa) became operational in 2008 and is based on the automatic retrieval of data directly from electronic patient dental records [17]. The total amount of dental care information (i.e. caries and periodontitis) includes 7.4 million people out of Sweden’s total population of approximately 10 million people [18]. Applying the previously mentioned method of describing longitudinal caries data as three different caries development trajectories to SKaPa data might be a first step to identify caries-active individuals and their experience of caries disease in the Swedish population. Investigation of longitudinal caries data for the individuals exhibiting the highest caries experience may also assist in identifying the actual caries–prevention treatment needs for this patient group and may assist in developing more effective preventive treatments and measures [19].

The current study aims to identify caries-active individuals among 30- and 40-year-olds by analyzing longitudinal general population-based data from SKaPa and to compare these results divided into three different caries development trajectories as described previously for the Dunedin cohort [4,7]. Our hypothesis is that the group with the highest caries experience, expressed as mean DMFS index, will be of similar proportions in the two different SKaPa age groups using the three-trajectory model when compared with the Dunedin data.

## Materials and methods

Longitudinal caries data from 30- and 40-year-old individuals in SKaPa collected over a 10-year observation period were compared with previously published data from the Dunedin studies [4,7].

Two age groups of patients (30 and 40-years old, regular attendees at general dental clinics) were selected from SKaPa in 2019. Longitudinal caries data were then retrieved for the previous 10 years (i.e. from 2010).

The numbers of included individuals from the two age groups were 43,490 (30-year-olds) and 22,681 (40-year-olds). The total sample from SKaPa thus included caries data for 66,171 individuals for the period 2010–2019.

The Dunedin longitudinal study of a birth cohort started with children born at the Queen Mary Hospital, Dunedin, New Zealand, between April 1, 1972, and March 31, 1973. The original cohort included 1,037 individuals, with follow-up dental examinations at the ages of 5, 9, 15, 18, 26, and 32 years [4] and at the age of 38 years (2010) [7]. Data from the Dunedin cohort (*n* = 935) were analyzed using a method called group-based trajectory modeling, which is a specialized application of finite mixture modeling and can simplify longitudinal data by identifying developmental trajectory groups on a likelihood basis. It approaches a set of individual trajectories by grouping those which closely resemble one another (using a probability function). Dealing with a small number of groups of trajectories is less complicated than analyzing several hundred individual trajectories [4,20]. Analysis that resulted in three trajectories of caries experience was reported previously for the Dunedin cohort [4,7]. In these studies, caries development over time was followed, originally expressed as three different trajectories: that is, high (15%; *n* = 144), moderate (45%; *n* = 427), and low (40%; *n* = 364).

The two age groups from SKaPa were analyzed by comparison with the results from the Dunedin study [4,7]. Each age group was divided into three different trajectories of mean DMFS values in 2019 (high, 15%; moderate, 45%; and low, 40%), similar to the trajectory model used in the Dunedin cohort [4,7].

The main observed variable was DMFS [21] over time for the included subjects, at baseline (2010) and at the end of the observation period (2019). The mean change in DMFS during the follow-up period constituted the main variable for describing the caries activity for each trajectory.

The definition used for the *D* component of the DMFS index used in SKaPa was defined as manifest caries located on any tooth surface, including secondary caries.

The registered decayed teeth (DT) and decayed tooth surfaces (DS) during the 10-year follow-up period was also compared for the different trajectories among the two age groups, expressed as DT and DS per year.

The Swedish Ethical Review Authority approved the current research project (Dnr 2022-01689-02).

### Statistics

Descriptive statistics were used regarding caries development during the follow-up period. Values were expressed as mean (including mean difference, *d*) ± standard deviation.

Similarities in caries development patterns were compared between the age groups and trajectories as well as with the data from the previously published Dunedin cohort.

Comparisons within groups were made using dependent sample *t* tests, and comparisons between groups were made using one-way analyses of variance (ANOVA) with Tukey– Kramer post hoc tests. Comparisons between trajectories and follow-up year were made using mixed-effects ANOVA. *p* values less than or equal to .01 were considered significant. Data were analyzed using IBM SPSS Statistics for Windows 28 (v; IBM Corporation, Armonk, NY, USA).

## Results

A total of 66,171 subjects from SKaPa were included in the study. The included individuals were regular recall patients from 1,333 dental clinics in Sweden. All included individuals were assigned to one of three caries trajectory groups based on their DMFS scores (i.e. high, moderate, and low caries experience) (Table 1). The mean DMFS at baseline (2010) and at the end of the study period (2019) was described for the three trajectories of the two age groups (30- and 40-year-olds) (Table 2).

**Table 1:** Number of subjects in the two age groups from the SKaPa registry aged 30- and 40 in 2019

	30 years old	40 years old
High (15%)	6237	3299
Moderate (45%)	20081	10619
Low (40%)	17172	8763
Total	43490	22681

The two age groups were divided into three caries trajectories according to the DMFS values in 2019. The observation period was 10 years, starting in 2010 and ending in 2019.

**Table 2:** Mean DMFS values at start (2010) and end (2019) of the observation period, in the two age groups from the SKaPa registry (30- and 40-year-olds) divided in three caries trajectories, respectively

Years	2010	2019		
DMFS	Mean ± SD	Mean ± SD	*d* ± *sd*	*p*
**30 years old**				
High (15%)	21.9 ± 9.7	29.8 ± 11.0	7.9 ± 10.6	<.001
Moderate (45%)	8.2 ± 5.4	10.5 ± 4.6	2.4 ± 4.2	<.001
Low (40%)	1.5 ± 2.9	1.6 ± 1.4	0.01 ± 2.7	*n.s.*
Total	7.5 ± 8.6	9.8 ± 10.6	2.2 ± 5.8	<.001
**40 years old**				
High (15%)	32.3 ± 13.2	41.7 ± 14.0	9.4 ± 11.6	<.001
Moderate (45%)	14.3 ± 6.2	17.8 ± 5.6	3.5 ± 4.4	<.001
Low (40%)	3.6 ± 3.1	4.4 ± 2.9	0.8 ± 2.3	<.001
Total	12.8 ± 11.7	16.1 ± 14.0	3.3 ± 6.2	<.001

DMFS = decayed, missing, and filled tooth surfaces.

**Figure 1:**
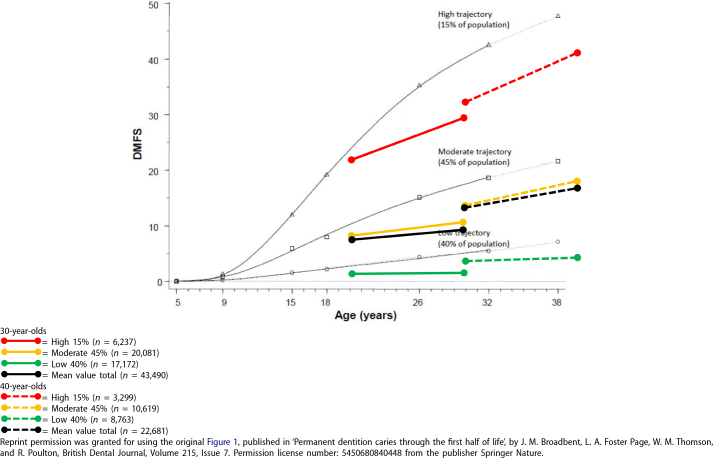
The original figure from the Dunedin cohort describing the three-trajectory model, High = 15% (*n* = 144), Moderate = 45% (*n* = 427) and Low = 40% (*n* = 364). Data from the two SKaPa cohorts (30-and 40-year-olds) described by the three-trajectory model (15%, 45% and 40% of the caries experience), including a total of 66 171 subjects. Dunedin data represented by the thin dark grey lines.

The mean increase in DMFS during the 10-year follow-up period was statistically significant for all three trajectories in both age groups, indicating that increase of DMFS occurred in all groups, however not significant in the low trajectory group in the youngest age group (Table 2). The increase was significantly elevated in both high trajectory groups compared with the other trajectories in the same age group, as shown by one-way ANOVA (*F*_5, 66165_ = 3385) and Tukey–Kramer post-hoc test (all *p* values < .001). The three different trajectories in the two age groups from SKaPa were compared with the Dunedin cohort (Figure 1).

To investigate the differences between the high trajectory and the moderate and low trajectory groups, two mixed-effects ANOVAs were calculated. For 30-year-olds, there was a main effect of trajectory (i.e. high, moderate, and low) (*F*_2, 43487_ = 61538) and time (2010 and 2019) (*F*_1, 43487_ = 14523), as well as an interaction (*F*_2, 43487_ = 5253), (*p* < .001). For the 40-year-old-group, there was also a main effect of trajectory (*F*_2, 22678_ = 34073) and time (*F*_1, 22678_ = 11892), and an interaction (*F*_2, 22678_ = 44584) (*p* < .001). Thus, for both age groups, the high trajectories differed significantly in relation to the moderate and low trajectories. The interactions indicate an elevated increase in DMFS for the higher trajectories compared with the moderate and low trajectories. The mean DT and DS values during the study period (2010–2019) for the three caries trajectories of the two SKaPa age groups are shown in Table 3. Mean DT and DS were analyzed using two one-way ANOVAs, with Tukey–Kramer post hoc tests. Mean DT and DS were significantly larger in the high trajectories compared with the other trajectories within the same age group (*F*_5, 66165_ = 2679, *p* < .001, and *F*_5, 66165_ = 2986, *p* < .001, respectively).

**Table 3:** Mean DS and mean DT values per year in the three caries trajectories of the two age groups from the SKaPa registry (30- and 40-year-olds) during the observation period 2010–2019.

	DS/year	DT/year
	Mean ± SD	Mean ± SD
**30 years old**		
High (15%)	0.45 ± 0.10	0.38 ± 0.09
Moderate (45%)	0.15 ± 0.04	0.14 ± 0.04
Low (40%)	0.03 ± 0.01	0.03 ± 0.01
**40 years old**		
High (15%)	0.39 ± 0.16	0.32 ± 0.12
Moderate (45%)	0.16 ± 0.06	0.14 ± 0.05
Low (40%)	0.05 ± 0.02	0.05 ± 0.02

DS = decayed surfaces; DT = decayed teeth.

The *p* values were statistically significant for the high trajectory vs the moderate and low trajectories among both 30-and 40-year-olds and for DS/year as well as DT/year.

The proportion of women was 54% (30-year-old group) and 55% (40-year-old group), respectively. There was an increase in the number of women in the higher caries trajectories for both age groups. The trajectory proportions in the 30-year-old group were: low trajectory, 52%; moderate trajectory, 53% and high trajectory, 57%. The corresponding proportions for the 40-year-old group were: low trajectory, 54%; moderate trajectory 55%; and high trajectory, 57%.

## Discussion

The method described by Broadbent and coworkers using different caries trajectories with longitudinal data from the Dunedin cohort has been shown to identify individuals with the highest caries experience [4,7]. The present study used this method in two Swedish nationwide age groups of 30-and 40-year-old individuals from SKaPa. The results were similar to the results of the Dunedin cohort regarding identification of the individuals with the highest caries experience during an observation period of 10 years.

The SKaPa data have been validated and demonstrate satisfactory reliability and accuracy regarding dental caries in 6- and 12-year-old children Thus, these data have been considered a reliable source for registry-based research [22]. Data registered in the dental record concerning diagnosis and treatment codes for adults were also retrieved automatically by SKaPa and the same data were delivered to the Swedish dental insurance system for financial reimbursement to dental organizations as well as to patients [23]. This dual function of diagnosis and treatment codes could be presumed to provide high data validity in the SKaPa groups.

When comparing the highest trajectories of the SKaPa groups and the Dunedin cohort, it must be taken into account that approximately 20% of the Swedish adult population do not attend dental care regularly and are thus not included in the SKaPa data [24]. It is well-known that these individuals frequently suffer from impaired dental health. The main reasons for not attending are related to dental fear [25–28], as well as socioeconomic or financial factors [29–31].

The mean DMFS is decreasing over time. This can be seen by a comparison of DMFS at the age of 30 years, in Figure 1, occurring in 2010 for the 40-year-old group and in 2019 for the 30-year-old group in the SkaPa data, and in 2002 for the Dunedin cohort. A possible explanation for this phenomenon could be a general decrease in caries prevalence as described in different countries [9–11].

The accumulated caries experience, defined as DMFS at baseline for the Swedish age groups, already revealed a statistically significant difference between the three trajectories at 20 years of age in the youngest SKaPa age group (Figure 1). This significant difference may indicate that caries disease starts at a young age [32–34] and continues into adulthood. Caries activity among young children increases the continuing risk for new caries over time. Alm and co-workers found that 10–15% of 15-year-old individuals could be considered as ‘high risk’ and that 10% of the 15-year-old individuals had 74% of all approximal fillings and manifest caries lesions [33]. This high risk continues at 20 years of age [34]. A similar finding was described by Hall–Scullin and coworkers, concluding that ‘caries-free and caries-active children should be considered as two separate populations, suggesting different prevention strategies are required to address their different risk profiles’ [32].

The ability to control the caries process is essential to break the restorative replacement spiral created by caries progression. Fillings are being replaced with more extensive fillings, ultimately leading to dental pulp involvement and endodontic treatment, followed by crown preparation and then replacement crowns, finally leading to loss of teeth [35].

The study of caries data from SKaPa revealed that 15% of the highest caries experience group demonstrated continuing caries disease. Extrapolating this limited caries experience data from 30- and 40-year-old individuals to the entire Swedish population would yield an estimate of over one million individuals suffering from recurring caries disease. Use of only total mean DMFS values for an entire population will potentially hide the caries experience and presumed treatment need of the highest trajectories, because they just express low total mean values (Figure 1).

The gender difference found in this study, with more women in the higher caries trajectories for both SKaPa age groups was not observed in the Dunedin cohort [4,7] and needs further investigation.

The method using the three-trajectory model with the same proportions as in the Dunedin study have limitations and will need further analyzes of alternative trajectory proportions.

The results from this study suggest a need to develop better and more efficient methods for caries prevention for the group with the highest caries experience to stop further caries progression in the future. Such an approach could save substantial amounts of money for individuals, for society, and for dental organizations. Furthermore, it could prevent inconvenient dental treatment, suffering, and pain for many patients.

A need for a more standardized method of classifying caries, with a focus on more than just the dentinal or cavitation stages of caries as a threshold for making the decision to treat (or fill), has been discussed [36]. Consequently, new management protocols including decisions for preventive care plans have been proposed when high caries risk is determined and active caries detected [37]. The development of more precise descriptions about what prevention methods should be involved are needed [38], to dissiminate effective simple interventions that may work for anyone, independently of personal experience. The SKaPa annual reports have described this need for adults that received dental restorations due to caries during 2016, 59% of whom had received new restorations due to caries within 2 years (*N* = 1, 350,598). It is noteworthy that less than 30% of the caries-active adults receiving new restorations had obtained any form of documented preventive treatment or information [39].

Longitudinal caries data for adults are absent in many countries. Thus, SKaPa provides a unique resource to investigate the prevalence of recurring caries disease. These results should be considered as promising, and a future prospect may be to use the three-trajectory model first described in the Dunedin cohort on more SKaPa age groups to determine whether similar patterns exist in other cohorts.

By using this trajectory model, the most caries-active patients in the study can be identified, but the model can also be used for other age ranges and thus give rise to specific preventive programs with the aim of reducing disease activity across age groups.

New knowledge can increase interest in the need for preventive measures. The strength of using a national register can give rise to new national initiatives for specific prevention efforts.

## Conclusions

Using the three-trajectory model for the presentation of caries experience over time may be a useful tool to identify individuals with different disease activities. Identification of individuals with the highest caries experience could increase the possibilities to explore and evaluate more effective caries–prevention methods for this group.

## Data Availability

The data are available on reasonable request to the corresponding author.
